# Early-life antibiotic exposure and type 1 diabetes risk: a systematic review and meta-analysis

**DOI:** 10.3389/fendo.2026.1764522

**Published:** 2026-02-13

**Authors:** Callum De Pasquale, Leonard C. Harrison

**Affiliations:** 1 Walter and Eliza Hall Institute of Medical Research, Parkville, VIC, Australia; 2 Department of Medical Biology, The University of Melbourne, Parkville, VIC, Australia

**Keywords:** antibiotic exposure, early life, meta-analysis, pre-conception, systematic review, type 1 diabetes

## Abstract

**Objective:**

Antibiotic exposure impacts the gut microbiome and potentially, in an infant, the developing immune system, with implications for the emergence of immune disorders such as type 1 diabetes (T1D). Reports of early-life antibiotic exposure on risk for T1D are inconsistent. We aimed to perform a systematic review and meta-analysis of the association between antibiotic exposure in early life and the development of T1D.

**Methods:**

Observational studies were assembled that reported an association between early-life antibiotic exposure and the development of T1D. Four early-life periods were covered: 12 months preconception, prenatal (in pregnancy), neonatal and up to 24 months postnatal.

Medline, Embase, Web of Science Core Collection, and Scopus were searched from inception to August 28, 2024. All records were imported into Covidence for automated deduplication, abstract screening and full-text screening by two independent reviewers.

Data from 20 studies and 10, 960 T1D cases were extracted and analysed using a random effects meta-analysis. Pooled odds ratios (ORs) and hazard ratios (HRs) with associated 95% confidence intervals (CIs) were calculated.

**Results:**

In the preconception period, maternal exposure to macrolide (OR = 1.23 [95% CI: 1.02–1.48]), sulfonamide/trimethoprim (OR = 1.34 [95% CI: 1.07–1.69]) or tetracycline (OR = 1.26 [95% CI: 1.11–1.44]) antibiotics was associated with an increased odds of T1D. Prenatal, neonatal and postnatal antibiotic exposure was not significantly associated with T1D.

**Conclusion:**

Preconception exposure to specific antibiotic classes may represent a modifiable maternal risk factor for T1D in the offspring. This would have implications for antibiotic prescribing guidelines but requires validation by the further study of defined antibiotic classes and their exact timing of preconception exposure.

**Systematic review registration:**

The protocol was pre-registered on PROSPERO (CRD42024589374) and followed PRISMA guidelines.

## Introduction

Type 1 diabetes (T1D) is a major disease of childhood, affecting over 1.5 million children worldwide, that is characterised by the progressive autoimmune destruction of insulin-producing β cells in the pancreatic Islets of Langerhans (1). T1D can result in acute metabolic disturbances such as hypoglycemia and ketoacidosis, and chronic cardiovascular, renal, retinal and neuronal complications. Along with the demands of daily management, these outcomes contribute to psychological stress and impaired quality of life. Additional to its burden on individuals and families, T1D imposes a substantial cost on the healthcare system (2). The incidence of T1D has been rising, due to changing environmental factors that increase penetrance of risk genes (3). While recent interventions with immune agents have slowed loss of β-cell function (4), long-term remission remains elusive. To alleviate the burden of disease it is important to focus on disease prevention and identify modifiable environmental risk factors.

Alterations in the gut microbiome have been described in T1D (5), including a decrease in bacterial taxonomic diversity, also seen after oral antibiotic administration. The connection with the gut microbiome underpins the proposition that early-life antibiotic exposure may be a potential environmental risk factor for T1D. This is supported by studies in the non-obese diabetic (NOD) mouse, a model of spontaneous autoimmune diabetes, which demonstrate that early-life antibiotic exposure influences the development of diabetes (6–8). Administration of vancomycin in drinking water from birth to weaning decreased the incidence of diabetes (6). Fecal bacterial diversity was decreased, leaving a single dominant species, *Akkermansia muciniphila*, and small intestinal proinflammatory CD4^+^ T cells were increased (6). In contrast, continuous exposure to vancomycin or neomycin from just before birth (to mothers) increased the incidence of diabetes (7). Again, ileal and colonic bacterial diversity were decreased with an increase in the abundance of *Akkermansia muciniphila*, as well as Enterobacteriaceae, and small intestinal proinflammatory CD4^+^ T cells were increased (7). In another study, the macrolide antibiotic tylosin tartrate, administered before and just after weaning, accelerated the onset of diabetes, especially in lower incidence males; the β-lactam, penicillin V, given at sub-therapeutic doses to mothers in pregnancy and offspring up to three months of age had no effect (8). These contrasting results suggest that the timing of antibiotic exposure influences the risk of T1D, pre-weaning being protective, in contrast to post-weaning.

Many cohort studies (9–28) have investigated an association between early-life antibiotic exposure and T1D risk but they have not been subjected to a comprehensive systematic review and meta-analysis. Here, we synthesise existing evidence in a systematic review and meta-analysis of the association between early-life antibiotic exposure and T1D. Because studies in the NOD mouse model suggest that timing of antibiotic exposure may be critical, the review encompassed four exposure windows: preconception (12 months before conception), prenatal (during pregnancy), neonatal (first two weeks after birth) and postnatal (0–6 months, 0–12 months, 0–24 months after birth). The following were of interest: any antibiotic, specific antibiotic classes (macrolides, sulfonamide/trimethoprim, tetracyclines) and antibiotic spectra (broad or narrow), as well as the number of antibiotic courses. Determining if antibiotic exposure in early life influences T1D risk could guide research on antibiotic-microbiota interactions in T1D and inform clinical decision-making about antibiotic use.

## Materials and methods

### Registration

The protocol for this systematic review and meta-analysis was pre-registered on PROSPERO (CRD42024589374) and follows PRISMA guidelines (**Supplementary File 1**).

### Information and search strategy

A systematic literature search was conducted in Medline, Embase, Web of Science Core Collection and Scopus from database inception to August 28, 2024. No language restrictions were applied. The search strategy was developed using key terms, MeSH terms, and Emtree terms, and was reviewed by librarians at the University of Melbourne. The full search strategies are available in **Supplementary File 2**.

### Eligibility criteria

Studies were eligible for inclusion if they met the following criteria:

1. Study design: cohort or case-control observational studies.
2. Population: pregnant women and children under two years of age.
3. Intervention: antibiotic exposure during early life (12 months preconception, prenatal (during pregnancy), neonatal (first two weeks after birth), postnatal (up to 24 months after birth).
4. Comparator: no antibiotic exposure.
5. Outcome: T1D diagnosis.

### Study selection and screening

All records retrieved from the database searches were imported into Covidence as Research Information Systems (RIS) files for automated deduplication, abstract screening and full-text screening. Two independent reviewers screened titles and abstracts for study relevance, followed by full-text assessment of studies. Conflicts at both the abstract and full-text screening stages were resolved through discussion. Only published studies were included. The RIS files are available in **Supplementary File 3**.

### Data collection and extraction

Data extraction was conducted by one reviewer. The following data were extracted from each study:

1. Study details: author, year, country, study design.
2. Population characteristics: number of cases with/without antibiotic exposure, percentage female.
3. Intervention details: period of antibiotic exposure, specific antibiotic classes, number of courses.
4. Data sources: source of antibiotic exposure data (e.g., prescription records, parental self-report).
5. Outcome details: T1D definition (e.g., clinical diagnosis, islet antibody positivity), age at outcome assessment.
6. Confounders: adjustments made (e.g., age, sex, mode of delivery).

### Statistical analysis

A random-effects model was used for meta-analysis, implemented with meta package in R. The primary effect measure was the odds ratio (OR) with 95% CIs. Some studies reported the hazard ratio (HR), which was analyzed separately and not pooled with ORs.

Heterogeneity was assessed using the I² statistic, τ², and p-value. Only the prenatal OR meta-analysis included heterogeneity measures, as it had the largest number of contributing studies (n = 8).

Subgroup analyses were conducted when at least two studies reported data on the same exposure and period. These included: any antibiotic use; specific antibiotic classes (e.g., macrolides, beta-lactams, quinolones); number of antibiotic courses (1–2 vs. ≥3); broad- vs. narrow-spectrum antibiotics.

## Results

### Study selection

**Figure 1:**
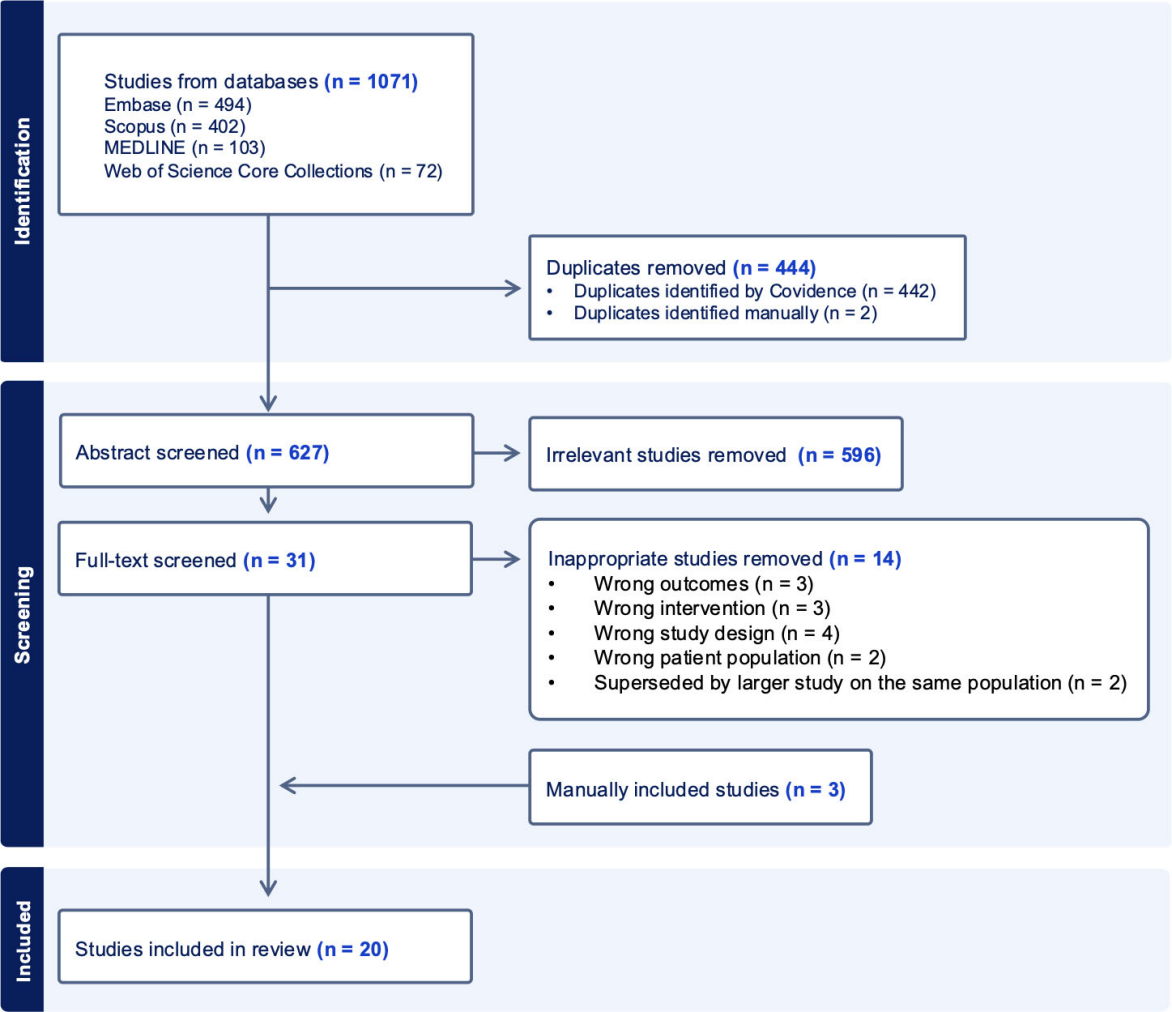
PRISMA diagram illustrating the study selection process for the systematic review and meta-analysis.

1, 071 records were retrieved across Embase, Scopus, Medline and Web of Science Core Collections. After automated duplicate removal in Covidence (n = 442) and manual removal of two additional duplicates, 627 unique records remained for abstract screening. Following abstract screening, 596 irrelevant records were excluded, leaving 31 studies for full-text screening. After full-text screening, 14 studies were excluded, resulting in 17 eligible studies. Additionally, 3 studies meeting the inclusion criteria were identified outside the search and included manually. Thus, 20 studies were included in the systematic review. The study selection process is illustrated in **Figure 1**.

### Features of included studies

The characteristics of the included studies are summarised in **Table 1**, with additional details available in the full data extraction table (**Supplementary File 4**). The 20 included studies spanned 14 countries, with the majority conducted in Europe (United Kingdom, Germany, Luxembourg, Latvia, Lithuania, Romania, Malta, Sweden, Finland, Denmark, Norway), alongside studies from Israel, South Korea and the USA. All studies were observational and examined the association between early-life antibiotic exposure and T1D risk. Collectively, they comprised over 3 million participants and 10, 960 T1D cases.

**Table 1:** Features of included studies.

Author	Year	Country	No cases: cohort size	Age (years) at diagnosis	Percentage female	Confounders accounted for
Blom (9)	1991	Sweden	339:867	0-14	47.2	Age, sex, country
McKinney (10)	1997	UK	196:521	<16	NR*	Age, sex
EURODIAB (11)	2000	Latvia Lithuania Luxemburg Romania England Northern Ireland	1028:4072	<15	NR	Age, site, breast feeding, birth weight, maternal age, jaundice at birth, asthma before disease diagnosis, vitamin D supplementation
Kilkkinen (12)	2006	Finland	437:2185	2.7 mean	50.1	Age, sex, hospital district
Cardwell (13)	2008	UK	367:4579	5.9 mean	54.5	Age, sex, region, non-infection related GP consultations
Hviid (14)	2009	Denmark	454:606420	4.4 mean	NR	Age, calendar period, maternal ethnicity
Virtanen (15)	2014	Finland	223:6242	<15	47.6	Sex, genetic risk (HLA-DQB1), family history, delivery mode, birthplace, parental asthma/allergic rhinitis, maternal education, maternal age, home municipality urbanization level, asthma/atopic eczema in the child by age 5 years
Mikkelsen (16)	2016	Denmark	250:2236	<16	49.8	Sex, age
Clausen (17)	2016	Denmark	1503:858201	<15	48.7	Sex, birth year, parity, delivery mode
Kemppainen (18)	2017	Finland, Germany, Sweden, USA	463:8495	<4.1	49	Sex, country, T1D/celiac disease family history, HLA-DR genotype, Caesarean delivery, probiotic use before age 90 days, breastfeeding, prenatal antibiotic use, season of birth
Haupt-Jørgensen (19)	2018	Denmark	336:75629	<18.4	NR	Maternal BMI, paternal BMI, maternal age, socioeconomic status, parity, maternal diabetes, smoking during pregnancy, birth weight, and gestational weight gain
Tapia (20)	2018	Norway	835:537458	4.4 mean	51	Sex, maternal age and parity, maternal T1D, prenatal smoking, education level, pre-pregnancy BMI, birthweight
Antvorskov (21)	2020	Denmark	NR:50931	<18.4	NR	Socioeconomic status, parity, maternal diabetes, smoking during pregnancy, delivery mode, breastfeeding
Wernroth (22)	2020	Sweden	1238:760907	4.2 mean	48.5	Sex, parity, prenatal smoking, maternal T1D, maternal age, parental birthplace, parental education, disposable income, birth year, birth season, region of residence, population density, maternal BMI, delivery mode, gestational age, paternal T1D, birth weight
Belteky (23)	2020	Sweden	126:14910	<18	47	Sex, T1D in the father, maternal autoimmune disease
Zargari (24)	2022	Israel	52:184	8.2 median	50	Maternal illness, birth weight, neonatal intravenous glucose infusion, neonatal feeding method
Abela (25)	2022	Malta	89:178	11 mean	46.1	Gestational age, birth weight, delivery mode, infant feeding, number of household siblings, parental smoking, parental age
Lee (26)	2022	South Korea	53:63434	<8	48.3	Age, sex, household income, and overweight
Raisanen (27)	2023	Finland	102:959	11 mean	40.2	Age, sex, residential area, gestational age, delivery mode
Hakola (28)	2024	Finland	2869:74263	5.2 mean	46	Sex, delivery mode, gestational age, birth weight

*NR = not reported.

Most studies adjusted their effect sizes for key confounders, most commonly age, sex, country and mode of delivery, as indicated in **Table 1**. Eleven studies did not report ORs for all exposures and periods, despite providing the necessary data to calculate these insights. In such cases, ORs and CIs were independently calculated using the data provided in the studies and are included in the meta-analysis. All independent calculations are distinguished in the extraction table (**Supplementary File 4**). These independently derived estimates do not account for additional confounders beyond those considered in the original matching of controls to cases.

The meta-analysis examined the association between early-life antibiotic exposure and T1D, with pooled estimates calculated for the four different exposure periods.

### Preconception (12 months preceding conception)

**Figure 2:**
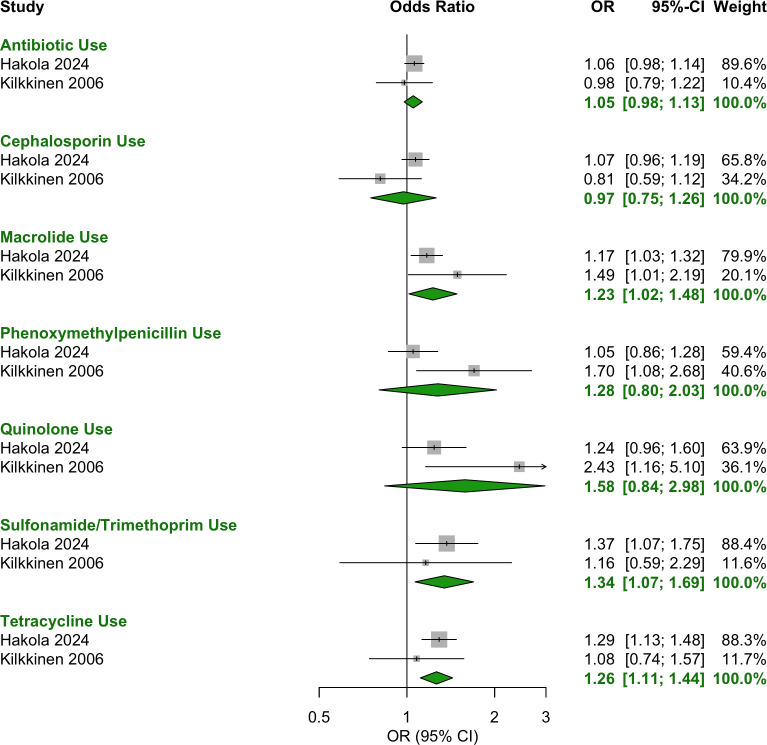
Forest plot of OR estimates and 95% CIs for the association between antibiotic use in the year before conception and T1D. Pooled estimates were calculated using a random-effects model. The diamond represents the pooled effect estimate, with its width indicating the 95% CI. Study weightings in the meta-analysis are shown in the far-right column and visually represented by the size of the squares.

For the 12 months preceding conception, exposure to the following antibiotics was significantly associated with increased odds of T1D (**Figure 2**):

- Macrolide: OR = 1.23 [95% CI: 1.02–1.48].
- Sulfonamide/trimethoprim: OR = 1.34 [95% CI: 1.07–1.69].
- Tetracycline: OR = 1.26 [95% CI: 1.11–1.44].

The following antibiotic categories showed no significant association with T1D:

- Any antibiotic use (class agnostic): OR = 1.05 [95% CI: 0.98–1.13].
- Cephalosporin: OR = 0.97 [95% CI: 0.75–1.26].
- Phenoxymethylpenicillin: OR = 1.28 [95% CI: 0.80–2.03].
- Quinolone: OR = 1.58 [95% CI: 0.84–2.98].

### Prenatal (during pregnancy)

**Figure 3:**
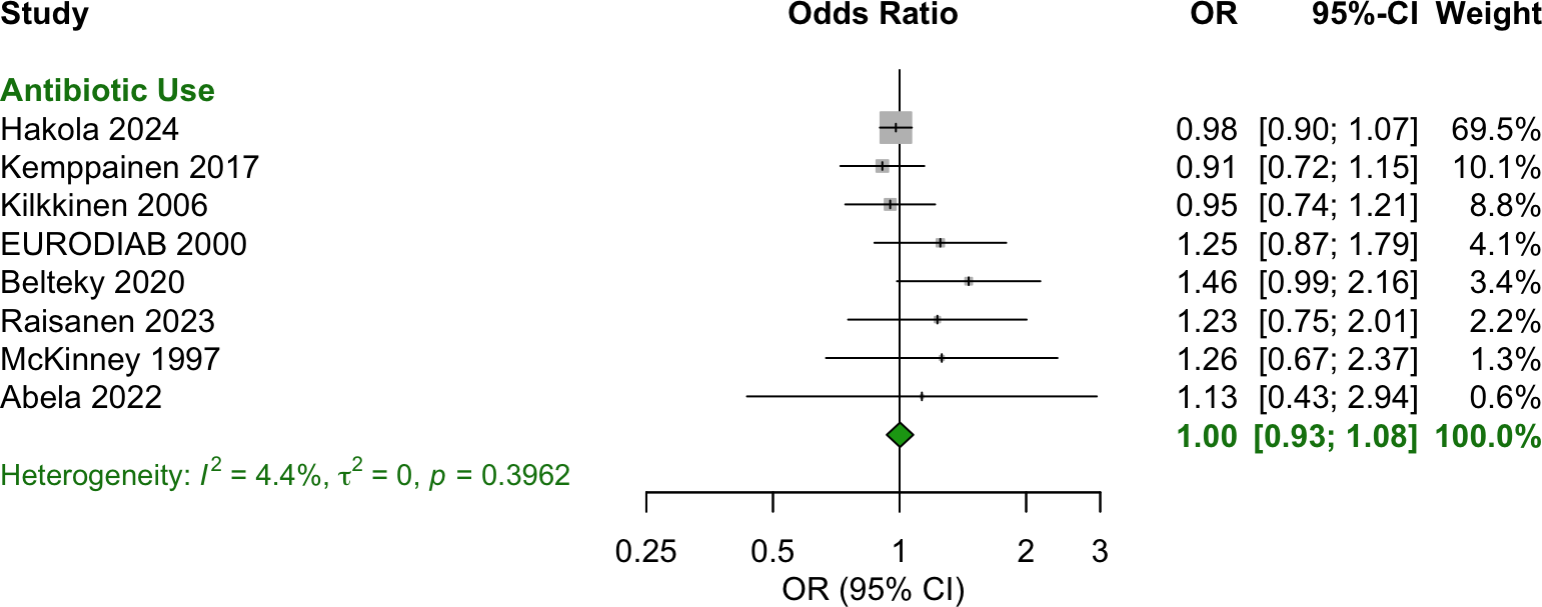
Forest plot of OR estimates and 95% CIs for the association between any antibiotic use prenatally and T1D.

**Figure 4:**
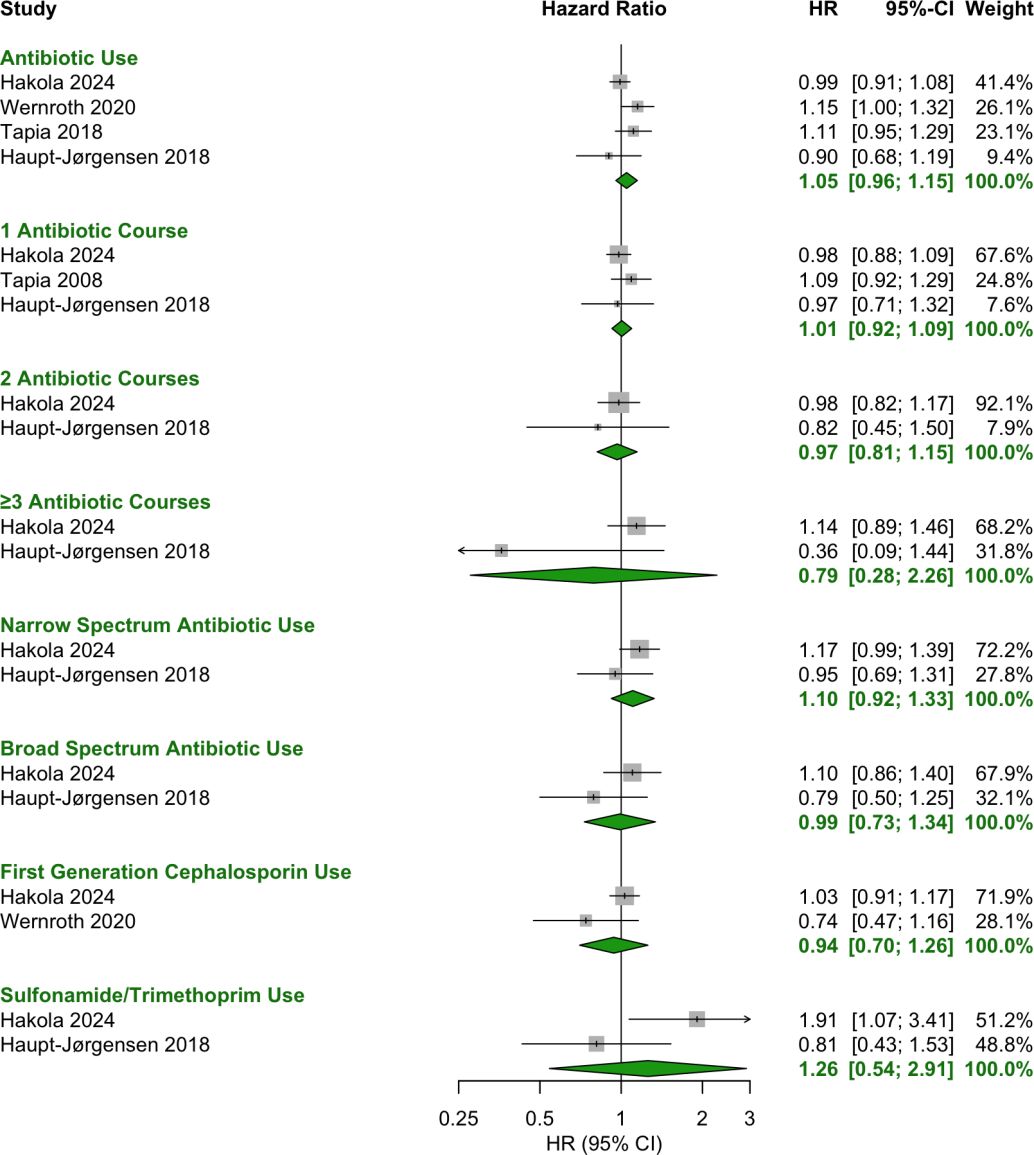
Forest plot of HR estimates and 95% CIs for the association between antibiotic use prenatally and T1D.

Any antibiotic exposure during the prenatal period showed no significant association with T1D (OR = 1.00 [95% CI: 0.93–1.08]) (**Figure 3**). Additionally, no specific antibiotic class or number of courses was associated with T1D (**Figure 4**).

### Neonatal

**Figure 5:**
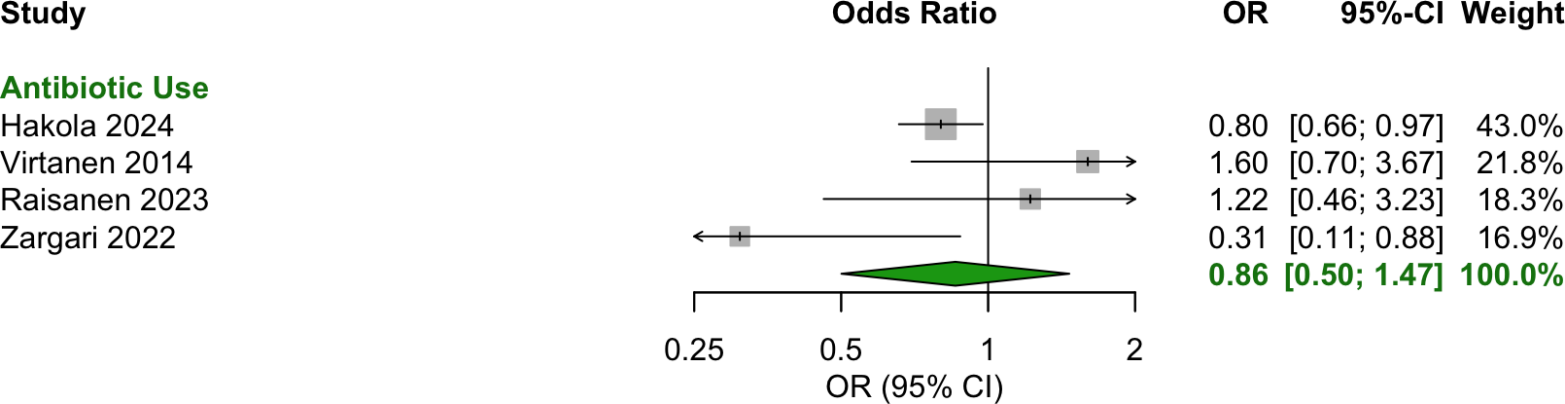
Forest plot of OR estimates and 95% CIs for the association between antibiotic use neonatally and T1D.

Any antibiotic exposure during the neonatal period showed no significant association with T1D (OR = 0.86 [95% CI: 0.50-1.47] (**Figure 5**). Data were inadequate to calculate pooled effect sizes specific for class, spectrum or number of courses.

### Postnatal (0–6, 0–12, 0–24 months postnatal)

No significant associations were observed for antibiotic use during the first two years of life, including in the periods 0–6 months (**Figure 6**), 0–12 months (**Figures 7**, **8**) and 0–24 months (**Figures 9**, **10**).

**Figure 6:**
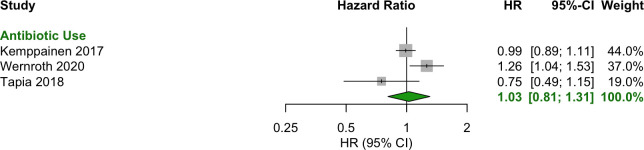
Forest plot of HR estimates and 95% CIs for the association between antibiotic use in the first 6 months after birth and T1D.

**Figure 7:**
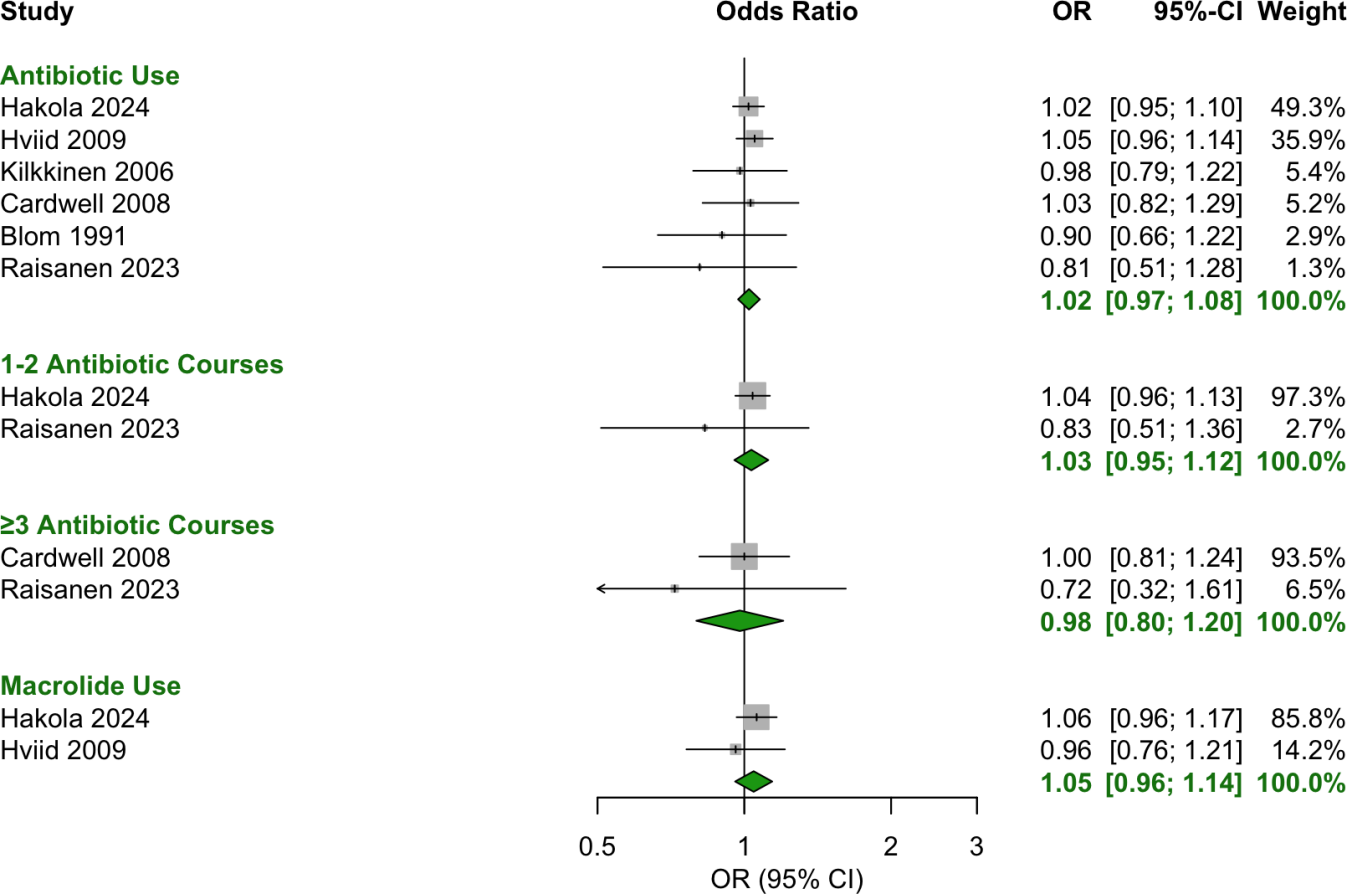
Forest plot of OR estimates and 95% CIs for the association between antibiotic use in the first 12 months of life and T1D.

**Figure 8:**
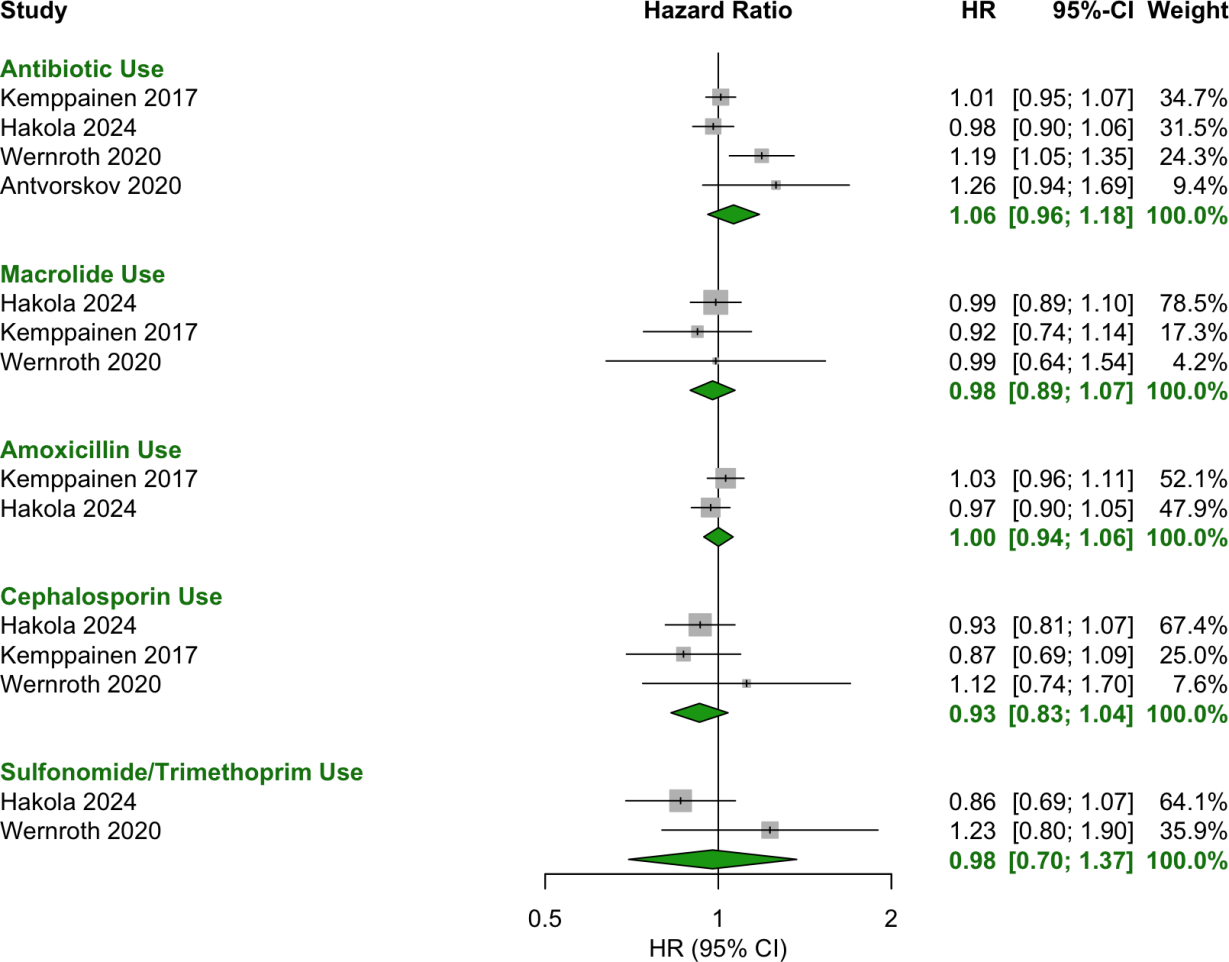
Forest plot of HR estimates and 95% CIs for the association between antibiotic use in the first 12 months of life and T1D.

**Figure 9:**
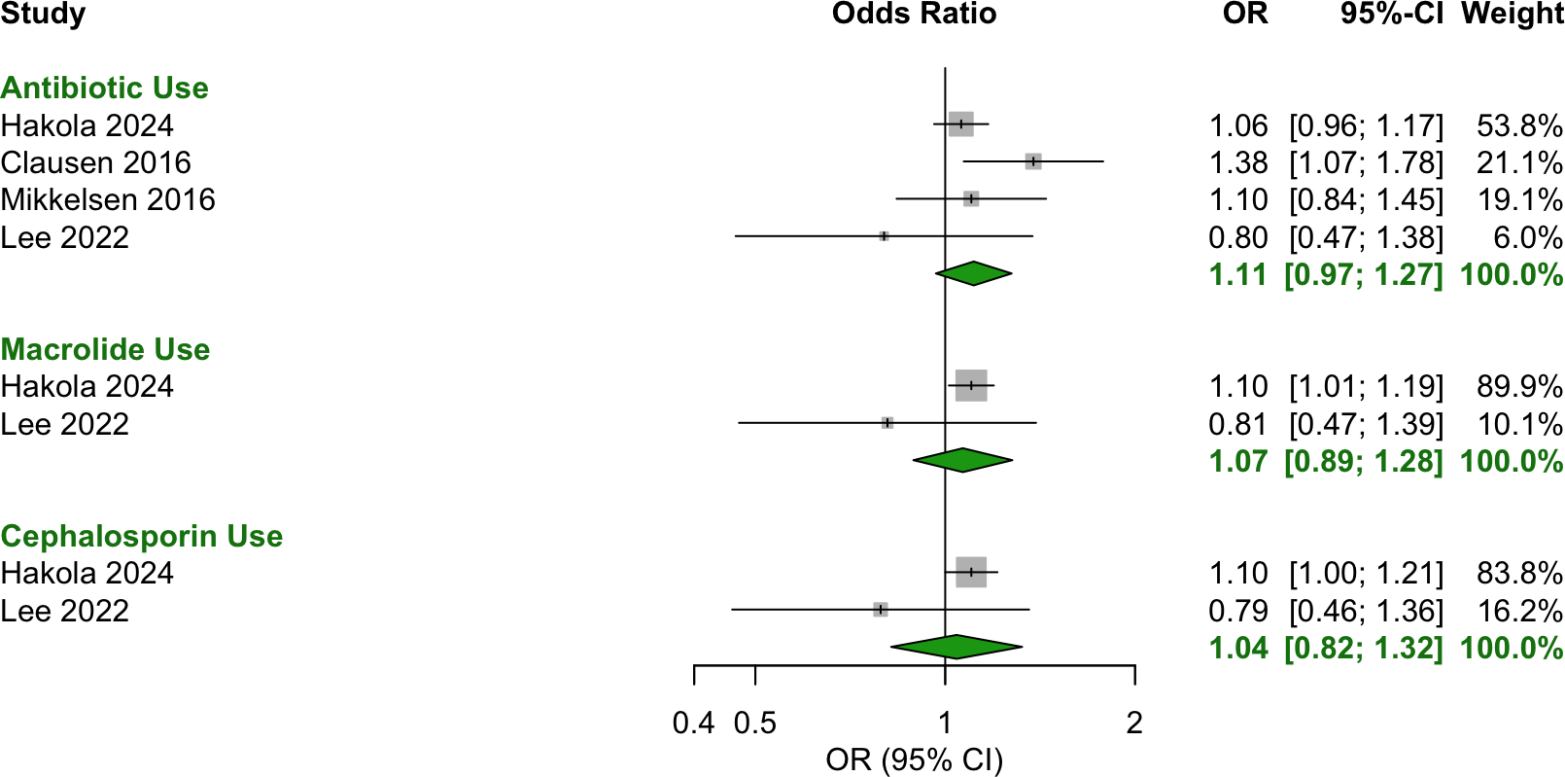
Forest plot of OR estimates and 95% CIs for the association between antibiotic use in the first 24 months of life and T1D.

**Figure 10:**
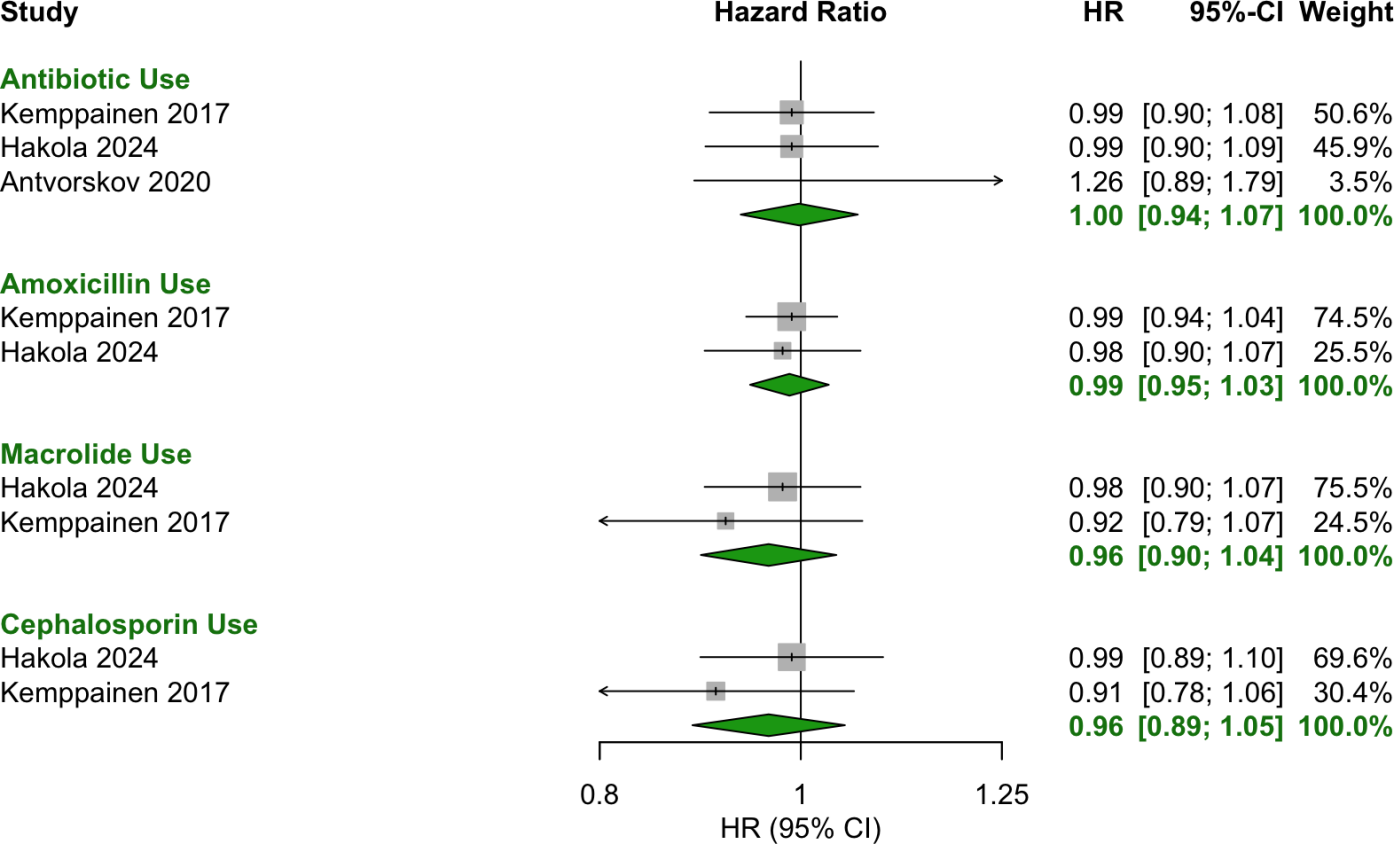
Forest plot of HR estimates and 95% CIs for the association between antibiotic use in the first 24 months of life and T1D.

### Heterogeneity assessment

Heterogeneity was most meaningfully assessed for the prenatal period, where eight studies (eight ORs and 95% CIs) contributed to the pooled estimate. The calculated heterogeneity statistics were I² = 4.4%, τ² = 0; p = 0.3962. These values suggest that the variability in effect sizes across studies is minimal, and differences in study results are likely due to random variation rather than systematic differences between the studies.

## Discussion

This meta-analysis found no evidence that exposure to antibiotics in pregnancy or early infant life was associated with the development of T1D. However, the odds of T1D development were increased by 23%, 34% and 26% after preconception exposure to macrolides, sulfonamides/trimethoprim and tetracyclines, respectively. In contrast, preconception exposure to any antibiotic (class agnostic) or cephalosporins was not associated with significantly altered odds. Similarly, phenoxymethylpenicillin and quinolone antibiotics showed no significant association, though their wide confidence intervals suggest substantial uncertainty. Across the two preconception studies, only 140 cases were exposed to phenoxymethylpenicillin and 77 to quinolones, limiting statistical power. A true association may exist but not be detected due to the small sample size.

The individual preconception studies provide context to these significant pooled results. In the Hakola 2024 study (28), preconception macrolide use was associated with both increased odds of T1D and increased rate, strengthening confidence in this association. In contrast, sulfonamides/trimethoprim and tetracyclines had significant ORs but non-significant adjusted HRs, weakening the certainty of their association with T1D. This discrepancy may stem from limited statistical power in the Hakola study, in which only 68 cases were exposed to sulfonamides/trimethoprim and 236 to tetracyclines, compared to 270 for macrolides. The meta-analysis increased the sample size from 68 to 79 for sulfonamides/trimethoprim and 236 to 275 for tetracyclines but lacks adjustment for confounders beyond case matching for age and sex, unlike the adjusted HRs in Hakola (28). Because the adjusted HRs in the Hakola study did not identify a significant association for preconception exposure to sulfonamides/trimethoprim or tetracyclines with T1D, larger, well-powered studies with robust confounder adjustment will be needed to clarify these relationships.

Given higher confidence of the association between preconception exposure to macrolides and T1D, it is relevant to consider possible mechanisms. Macrolides have rapid and profound effects on intestinal microbiome diversity and composition that may persist for many months, especially when administered early in life. The more consistent effects include a decrease in alpha diversity and in the beneficial taxa *Bifidobacteria, Lactobacilli* and *Akkermansia muciniphila* spp that produce anti-inflammatory short chain fatty acids (29–31). These ‘dysbiotic’ alterations are similar to those reported in the fecal microbiome of children with T1D (32). In addition, macrolides have anti-inflammatory and immunomodulatory properties independent of their bacteriostatic effects (33, 34). That macrolides could have anti-inflammatory effects on the maternal gut microbiome to increase the risk for T1D in offspring appears counterintuitive. However, it is widely accepted that immune activation is necessary to induce maturation of immune regulatory pathways that protect against autoimmune disease. In the non-obese diabetic (NOD) mouse model of T1D, immune activation by mycobacterial adjuvant (35) or Toll-like receptor agonists (36) promotes immune regulation that protects against diabetes. In humans, females with T1D have a significantly lower risk of having a child with T1D than males with T1D (37). One explanation for this ‘maternal protection’ may be promotion of immune regulation by the pro-inflammatory state of the maternal gut microbiome in pregnancy in women with T1D (38). We suggest therefore that macrolides could offset protection against autoimmunity afforded by specific gut microbes at an early stage of development. Different macrolides have common and type-specific effects on the gut microbiome. However, the preconception studies in this review did not categorise macrolides by type (e.g. azithromycin, clarithromycin, erythromycin, roxithromycin) or time of exposure within the 0–12 months before conception.

Despite the inclusion of large, cross-country cohorts and adherence to strict eligibility criteria, several caveats limit the applicability of our findings. First, antibiotic exposure characteristics were often incomplete. Exposure windows were broad. For example, the 0-12-month preconception period may have included periods that are irrelevant to T1D risk. Broad exposure windows may dilute meaningful signals and contribute to statistically non-significant findings, despite the presence of a true effect in a critical subperiod. Although the effects of macrolides on the gut microbiome may last many months, it is plausible that a narrower window before conception would be biologically more relevant. Second, the measurement of antibiotic exposure varied. The Hakola study (28) relied on prescription reimbursement records, which do not consider hospital- administered antibiotics. Similarly, the Kilkkinen study (12) used a nationwide drug prescription register, which does not consider actual consumption or unprescribed use. Nevertheless, both studies found significant positive associations between preconception macrolide exposure and T1D. Other studies used parental questionnaires, which are prone to recall bias. Both register- and questionnaire-based approaches risk exposure misclassification, potentially resulting in antibiotic-exposed individuals being included in the control group or unexposed individuals in the case group. Misclassification could shift estimates towards the null. Third, race/ethnicity could impact the interpretation and generalisability of the findings, especially as the risk of T1D is higher in individuals of Northern European descent who carry specific human leukocyte antigen (HLA) haplotypes (1). However, analysis of the influence of race/ethnicity was not feasible because minimal data were reported in the included studies. Also, the two preconception studies were both Finnish register-based, with potentially overlapping samples in a high-risk T1D population. Future studies from other countries and more diverse populations would strengthen validity for this exposure window. Finally, studies were observational in design and cannot address causality. In addition, as in many studies, potential publication bias, where null findings are less likely to be published and hence included in a review, may inaccurately shift results towards false significance. Taken together, these caveats indicate that the evidence can be accepted with only moderate certainty and should be strengthened by further studies.

Other limitations are related to the review process. First, only four literature databases were searched (Medline, Embase, Web of Science Core Collection, and Scopus). While these are major biomedical resources, other repositories (e.g., CINAHL, Global Health) may contain relevant studies not identified. Second, data extraction was performed by a single reviewer, increasing the risk of transcription errors. Third, no formal risk of bias assessment (e.g. ROBINS-I) was conducted beyond applying predefined inclusion and exclusion criteria.

In conclusion, the significant association between preconception exposure to macrolides, sulfonamides/trimethoprim and tetracyclines and the development of T1D suggests that exposure to these antibiotics in the preconception period may be a modifiable risk factor for T1D. However, given the moderate quality of the evidence, further well-powered, confirmatory studies are needed to inform clinical decision-making and public health policy. These would ideally explore narrower preconception exposure periods, e.g., 0–3 and 3–6 months, and document antibiotic subclasses. Prescription data, pharmacy dispensing records and self-reported adherence logs should be accessed where possible to minimize misclassification of cases and controls. Additionally, analysis of the gut metagenome of antibiotic-exposed and -unexposed women may provide insights into how disruption of maternal microbial communities could contribute to T1D risk in the offspring. The current review and meta-analysis can readily be adjusted to account for the results of future studies.

## Data Availability

The original contributions presented in the study are included in the article/**Supplementary Material**. Further inquiries can be directed to the corresponding author.
