# Supplementary material for: Early-life antibiotic exposure and type 1 diabetes risk: a systematic review and meta-analysis
Source: Front Endocrinol (Lausanne). 2026 Feb 13;17:1764522. doi: 10.3389/fendo.2026.1764522 (PMC12945788; doi:10.3389/fendo.2026.1764522)
Supplement: Supplementary file 1 [file DataSheet1.pdf]

eTable 1. Search strategy

Search	Keywords	Hits
**Ovid MEDLINE(R) Epub Ahead of Print and In-Process, In-Data-Review & Other Non-Indexed Citations and Daily <August 26, 2024>**		
1	Pregnant Women/ or Pregnancy/ or Pregnancy Outcome/ or Pregnancy, high-risk/ or exp Pregnancy Trimesters/ or Pregnancy Complications/ or Pregnancy Complications, Infectious/ or exp Pregnancy in Diabetics/ or Mothers/ or Maternal Exposure/ or Prenatal Care/ or exp Infant/	2111879
2	(pregnan* or gesta* or prenatal* or "pre-natal*" or antenatal* or "ante-natal*" or perinatal* or "peri-natal*" or postnatal* or "post-natal*" or trimester* or f?etus* or f?etal or neonat* or newborn* or infan* or baby* or babies* or "early life").mp. [mp=title, book title, abstract, original title, name of substance word, subject heading word, floating sub-heading word, keyword heading word, organism supplementary concept word, protocol supplementary concept word, rare disease supplementary concept word, unique identifier, synonyms, population supplementary concept word, anatomy supplementary concept word]	2799886
3	1 or 2	2820372
4	exp Anti-Bacterial Agents/	845504
5	(Antibiotic* or "Anti-biotic*" or Antibacteria* or "Anti-bacteria*" or Acetylspiramycin* or Aclacinomycin* or Aclarubicin* or Aculeacin* or Afabicin* or Alafosfalin* or Alalevonadifloxacin* or Alamethicin* or Allicin* or Amfomycin* or Amikacin* or Aminoglycoside* or "Aminopenicillanic acid*" or Aminopenicillin* or Aminosalicylic Acid* or Amoxicillin* or Amrubicin* or Anisomycin* or Annamycin* or Ansamycin* or Antofloxacin* or Aplasmomycin* or Aplysianin* or Asukamycin* or Avibactam* or Avilamycin* or Azithromycin* or Azlocillin* or Aztreonam* or Azurocidin* or Bacampicillin* or Bacitracin* or Bafilomycin* or Balhimycin* or Balofloxacin* or Beauvericin* or Bedaquiline* or Bersiporocin* or "Beta-lactam*" or Betamipron* or Bialaphos* or Biapenem* or Bicozamycin* or Bifonazole* or Bleomycin* or Boromycin* or Borrelidin* or Brefeldin* or Brilacidin* or Calcimycin* or Carbacephem* or Carbapenem* or Carbenicillin* or Carindacillin* or Carumonam* or Cefacetrile* or Cefaclor* or Cefadroxil* or Cefalexin* or Cefaloridine* or Cefalotin* or Cefamandole* or Cefapirin* or Cefatrizine* or Cefazedone* or Cefazolin* or Cefcapene* or Cefcanel* or Cefditoren* or Cefepime* or Cefetamet* or Cefiderocol* or Cefixime* or Cefmenoxime* or Cefmetazole* or Cefminox* or Cefodizime* or Cefonicid* or Cefoperazone* or Ceforanide* or Cefoselis* or Cefotaxime* or Cefotetan* or Cefotiam* or Cefoxitin* or Cefpirome* or Cefpiramide* or Cefpodoxime* or Cefprozil* or Cefquinome* or Cefradine* or Cefroxadine* or Cefsulodin* or Ceftaroline* or Ceftazidime* or Cefteram* or Ceftibuten* or Ceftiofur* or Ceftizoxime* or Ceftobiprole* or Ceftolozane* or Ceftriaxone* or Cefuroxime* or Cefuzonam* or Cefozopran* or Cephacetrile* or Cephalosporin* or Cephalothin* or Cephamycin* or Cephradine* or Chloramphenicol* or Chlortetracycline* or Cilastatin* or Ciprofloxacin* or "Clavulanic Acid*" or Clindamycin* or Clofazimine* or Clarithromycin* or Cloxacillin* or Concanamycin* or Contezolid* or Cyclacillin* or Cycloheximide* or Dactinomycin* or Dalbavancin* or Dalfopristin* or Danofloxacin* or Daunomycin* or Daunorubicin* or Demeclocycline* or Delafloxacin* or Delamanid* or Deoxydoxorubicin* or Desmycosin* or Dibekacin* or Dichloramine* or Dicloxacillin* or Difloxacin* or Dihydrostreptomycin* or Dirithromycin* or Dioxidine* or Doripenem* or Doxycycline* or Durlobactam* or Echinomycin* or Edeine* or Enmetazobactam* or Enniatin* or Enoxacin* or Eperezolid* or Epiroprim* or Eravacycline* or Erythromycin* or Erythronolide* or	1208848

	Ethambutol* or Ethionamide* or Everninomicin* or Exebacase* or Fidaxomicin* or Filipin* or Finafloxacin* or Fleroxacin* or Floxacillin* or Fosfomycin* or Framycetin* or Fusafungine* or Fusidate sodium* or Fusidic Acid* or Funobactam* or Garenoxacin* or Gatifloxacin* or Gemifloxacin* or Gepotidacin* or Gramicidin* or Habekacin* or Heliomycin* or Hygromycin* or Ibezapolstat* or Iclaprim* or Idarubicin* or Ikarugamycin* or Imipenem* or Isopenicillin* or Josamycin* or Kanamycin* or Ketolide* or Lactivicin* or Lasalocid* or Lascufloxacin* or Lefamulin* or Leucomycin* or Levonadifloxacin* or Levofloxacin* or Lincosamide* or Linezolid* or Lincomycin* or Lomefloxacin* or Lysobactin* or Lysostaphin* or Lymecycline* or Mafenide* or Malyngolide* or Manumycin* or Mecillinam* or Meropenem* or Mersacidin* or Metampicillin* or Methacycline* or Methicillin* or Mepartricin* or Mertansine* or Metronidazole* or Milbemycin* or Minocycline* or Miraxid* or Modithromycin* or Moxalactam* or Moxifloxacin* or Mupirocin* or Mureidomycin* or Murepavadin* or Myxothiazol* or Nafcillin* or Nafithromycin* or "Nalidixic Acid*" or Nebramycin* or Nemonoxacin* or Nemorubicin* or Neomycin* or Neosporin* or Netilmicin* or Nisin* or Nitrofurantoin* or Novobiocin* or Ofloxacin* or Oleandomycin* or Oligomycin* or Omadacycline* or Oritavancin* or Oxacillin* or "Oxolinic Acid*" or Oxytetracycline* or Pardaxin* or Paromomycin* or Patulin* or Pazufloxacin* or Peceleganan* or Pefloxacin* or Penicillin* or Pheneticillin* or Pirarubicin* or Piperacillin* or Pivampicillin* or Pivmecillinam* or Platensimycin* or Plazomicin* or Pleuromutilin* or Pradimicin* or Pretomanid* or Pristinamycin* or Prulifloxacin* or Pyrazinamide* or Pyrrolinodoxorubicin* or Quinupristin* or Quinacillin* or Ramoplanin* or Ranalexin* or Ranbezolid* or Ravtansine* or Relebactam* or Retapamulin* or Rifabutin* or Rifampin* or Rifampicin* or Rifamycin* or Rifaximin* or Roxithromycin* or Ruboxyl* or Sabarubicin* or Saframycin* or Salinomycin* or Sarecycline* or Selamectin* or Simocyclinone* or Sitafloxacin hydrate* or Soravtansine* or Squalamine* or Streptogramin* or Streptomycin* or Streptothricin* or Sulbactam* or Sulbenicillin* or Sulfacetamide* or Sulfadiazine* or Sulfamerazine* or Sulfamethoxazole* or Sulfamethoxypyridazine* or Sulfanilamide* or Sulfathiazole* or Sultamicillin* or Surfactin* or Tazobactam* or Tebipenem* or Telithromycin* or Temocillin* or Tetracycline* or Ticarcillin* or Tigecycline* or Tinidazole* or Tomopenem* or Trichostatin* or Trimethoprim* or Troleandomycin* or Tunicamycin* or Ureidopenicillin* or Valrubicin* or Vancomycin* or Vaborbactam* or Zorubicin*).mp. [mp=title, book title, abstract, original title, name of substance word, subject heading word, floating sub-heading word, keyword heading word, organism supplementary concept word, protocol supplementary concept word, rare disease supplementary concept word, unique identifier, synonyms, population supplementary concept word, anatomy supplementary concept word]	
6	4 or 5	1343191
7	3 and 6	104313
8	Diabetes Mellitus, Type 1/	88812
9	("type I diabet*" or "type 1 diabet*" or "type one diabet*" or T1D or "juvenile adj2 diabet*" or "p?ediatric diabet*" or "child* adj2 diabet*" or "insulin dependent diabet*" or IDDM or insulitis or "autoimmune diabet*" or "beta cell adj3 auto*" or "beta cell adj3 destruct*" or "beta cell adj3 deplet*" or "beta cell adj3 loss" or "islet adj3 destruct*" or "islet adj3 auto*" or "insulin adj3 auto*" or "insulin adj3 antibod*" or "insulin adj2 deplet*" or "insulin adj2 deficien*" or "insulin adj2 lack*" or "glutamic acid decarboxylase*" or "antiglutamic acid decarboxylase*" or "Glutamate Decarboxylase*" or "antiglutamate Decarboxylase*" or GAD or antiGAD* or GADA or GAD65* or antiGAD65* or "islet antigen 2*" or "IA-2*" or IA2* or IAA or ICA512* or "ICA-512*" or "Tyrosine Phosphatase-like Protein adj3 Auto*" or "Insulinoma Associated 2 adj3 Auto*" or "Zinc Transporter 8 adj2 Auto*" or "Zinc Transporter 8 adj2 antibod*" or ZnT8* or "pancre* adj2 auto*" or	123879

	"pancre* adj2 antibod*").mp. [mp=title, book title, abstract, original title, name of substance word, subject heading word, floating sub-heading word, keyword heading word, organism supplementary concept word, protocol supplementary concept word, rare disease supplementary concept word, unique identifier, synonyms, population supplementary concept word, anatomy supplementary concept word]	
10	8 or 9	154540
11	Risk/ or Risk Assessment/ or Risk Factors/ or Odds Ratio/ or Incidence/ or Prevalence/	1791235
12	(risk* or odds or ratio or likelihood* or probabil* or chance* or incidence* or prevalen* or rate* or suscept* or vulnerab* or propensit* or associat*).mp. [mp=title, book title, abstract, original title, name of substance word, subject heading word, floating sub-heading word, keyword heading word, organism supplementary concept word, protocol supplementary concept word, rare disease supplementary concept word, unique identifier, synonyms, population supplementary concept word, anatomy supplementary concept word]	12593350
13	11 or 12	12593350
14	Infant/ or exp Child/ or Child Health/ or Child Development/ or Adolescent/ or Adolescent Health/ or Adolescent Development/ or National Longitudinal Study of Adolescent Health/ or Pediatrics/	3754119
15	(child* or baby* or babies* or infan* or toddler* or p?ediatric or preteen* or teen* or adolescen* or "school-age*").mp. [mp=title, book title, abstract, original title, name of substance word, subject heading word, floating sub-heading word, keyword heading word, organism supplementary concept word, protocol supplementary concept word, rare disease supplementary concept word, unique identifier, synonyms, population supplementary concept word, anatomy supplementary concept word]	4780238
16	14 or 15	4788283
17	10 and 13 and 16	25987
18	7 and 17	103
	**Embase Classic+Embase <1947 to 2024 August 26>**	
1	Pregnant Woman/ or Pregnancy/ or First Trimester Pregnancy/ or Second Trimester Pregnancy/ or Third Trimester Pregnancy/ or High Risk Pregnancy/ or Pregnancy Complication/ or Maternal Diabetes Mellitus/ or Pregnancy in Diabetics/ or Pregnancy Outcome/ or Mother/ or Maternal Exposure/ or Prenatal Care/ or Infant/ or Baby/ or Newborn/	2295384
2	(pregnan* or gesta* or prenatal* or "pre-natal*" or antenatal* or "ante-natal*" or perinatal* or "peri-natal*" or postnatal* or "post-natal*" or trimester* or f?etus* or f?etal or neonat* or newborn* or infan* or baby* or babies* or "early life").mp. [mp=title, abstract, heading word, drug trade name, original title, device manufacturer, drug manufacturer, device trade name, keyword heading word, floating subheading word, candidate term word]	3323279
3	1 or 2	3370525
4	exp Antibiotic Agent/	2047427
5	(Antibiotic* or "Anti-biotic*" or Antibacteria* or "Anti-bacteria*" or	2135628

	Acetylspiramycin* or Aclacinomycin* or Aclarubicin* or Aculeacin* or Afabicin* or Alafosfalin* or Alalevonadifloxacin* or Alamethicin* or Allicin* or Amfomycin* or Amikacin* or Aminoglycoside* or "Aminopenicillanic acid*" or Aminopenicillin* or Aminosalicylic Acid* or Amoxicillin* or Amrubicin* or Anisomycin* or Annamycin* or Ansamycin* or Antofloxacin* or Aplasmomycin* or Aplysianin* or Asukamycin* or Avibactam* or Avilamycin* or Azithromycin* or Azlocillin* or Aztreonam* or Azurocidin* or Bacampicillin* or Bacitracin* or Bafilomycin* or Balhimycin* or Balofloxacin* or Beauvericin* or Bedaquiline* or Bersiporocin* or "Beta-lactam*" or Betamipron* or Bialaphos* or Biapenem* or Bicozamycin* or Bifonazole* or Bleomycin* or Boromycin* or Borrelidin* or Brefeldin* or Brilacidin* or Calcimycin* or Carbacephem* or Carbapenem* or Carbenicillin* or Carindacillin* or Carumonam* or Cefacetrile* or Cefaclor* or Cefadroxil* or Cefalexin* or Cefaloridine* or Cefalotin* or Cefamandole* or Cefapirin* or Cefatrizine* or Cefazedone* or Cefazolin* or Cefcapene* or Cefcanel* or Cefditoren* or Cefepime* or Cefetamet* or Cefiderocol* or Cefixime* or Cefmenoxime* or Cefmetazole* or Cefminox* or Cefodizime* or Cefonicid* or Cefoperazone* or Ceforanide* or Cefoselis* or Cefotaxime* or Cefotetan* or Cefotiam* or Cefoxitin* or Cefpirome* or Cefpiramide* or Cefpodoxime* or Cefprozil* or Cefquinome* or Cefradine* or Cefroxadine* or Cefsulodin* or Ceftaroline* or Ceftazidime* or Cefteram* or Ceftibuten* or Ceftiofur* or Ceftizoxime* or Ceftobiprole* or Ceftolozane* or Ceftriaxone* or Cefuroxime* or Cefuzonam* or Cefozopran* or Cephacetrile* or Cephalosporin* or Cephalothin* or Cephamycin* or Cephradine* or Chloramphenicol* or Chlortetracycline* or Cilastatin* or Ciprofloxacin* or "Clavulanic Acid*" or Clindamycin* or Clofazimine* or Clarithromycin* or Cloxacillin* or Concanamycin* or Contezolid* or Cyclacillin* or Cycloheximide* or Dactinomycin* or Dalbavancin* or Dalfopristin* or Danofloxacin* or Daunomycin* or Daunorubicin* or Demeclocycline* or Delafloxacin* or Delamanid* or Deoxydoxorubicin* or Desmycosin* or Dibekacin* or Dichloramine* or Dicloxacillin* or Difloxacin* or Dihydrostreptomycin* or Dirithromycin* or Dioxidine* or Doripenem* or Doxycycline* or Durlobactam* or Echinomycin* or Edeine* or Enmetazobactam* or Enniatin* or Enoxacin* or Eperezolid* or Epiroprim* or Eravacycline* or Erythromycin* or Erythronolide* or Ethambutol* or Ethionamide* or Everninomicin* or Exebacase* or Fidaxomicin* or Filipin* or Finafloxacin* or Fleroxacin* or Floxacillin* or Fosfomycin* or Framycetin* or Fusafungine* or Fusidate sodium* or Fusidic Acid* or Funobactam* or Garenoxacin* or Gatifloxacin* or Gemifloxacin* or Gepotidacin* or Gramicidin* or Habekacin* or Heliomycin* or Hygromycin* or Ibezapolstat* or Iclaprim* or Idarubicin* or Ikarugamycin* or Imipenem* or Isopenicillin* or Josamycin* or Kanamycin* or Ketolide* or Lactivicin* or Lasalocid* or Lascufloxacin* or Lefamulin* or Leucomycin* or Levonadifloxacin* or Levofloxacin* or Lincosamide* or Linezolid* or Lincomycin* or Lomefloxacin* or Lysobactin* or Lysostaphin* or Lymecycline* or Mafenide* or Malyngolide* or Manumycin* or Mecillinam* or Meropenem* or Mersacidin* or Metampicillin* or Methacycline* or Methicillin* or Mepartricin* or Mertansine* or Metronidazole* or Milbemycin* or Minocycline* or Miraxid* or Modithromycin* or Moxalactam* or Moxifloxacin* or Mupirocin* or Mureidomycin* or Murepavadin* or Myxothiazol* or Nafcillin* or Nafithromycin* or "Nalidixic Acid*" or Nebramycin* or Nemonoxacin* or Nemorubicin* or Neomycin* or Neosporin* or Netilmicin* or Nisin* or Nitrofurantoin* or Novobiocin* or Ofloxacin* or Oleandomycin* or Oligomycin* or Omadacycline* or Oritavancin* or Oxacillin* or "Oxolinic Acid*" or Oxytetracycline* or Pardaxin* or Paromomycin* or Patulin* or Pazufloxacin* or Peceleganan* or Pefloxacin* or Penicillin* or Pheneticillin* or Pirarubicin* or Piperacillin* or Pivampicillin* or Pivmecillinam* or Platensimycin* or Plazomicin* or Pleuromutilin* or Pradimicin* or Pretomanid* or Pristinamycin* or Prulifloxacin* or Pyrazinamide* or Pyrrolinodoxorubicin* or Quinupristin* or Quinacillin* or Ramoplanin* or Ranalexin* or Ranbezolid* or Ravtansine* or Relebactam* or Retapamulin* or Rifabutin* or Rifampin* or Rifampicin* or Rifamycin* or Rifaximin* or Roxithromycin* or Ruboxyl* or Sabarubicin* or	

	Saframycin* or Salinomycin* or Sarecycline* or Selamectin* or Simocyclinone* or Sitafloxacin hydrate* or Soravtansine* or Squalamine* or Streptogramin* or Streptomycin* or Streptothricin* or Sulbactam* or Sulbenicillin* or Sulfacetamide* or Sulfadiazine* or Sulfamerazine* or Sulfamethoxazole* or Sulfamethoxypyridazine* or Sulfanilamide* or Sulfathiazole* or Sultamicillin* or Surfactin* or Tazobactam* or Tebipenem* or Telithromycin* or Temocillin* or Tetracycline* or Ticarcillin* or Tigecycline* or Tinidazole* or Tomopenem* or Trichostatin* or Trimethoprim* or Troleandomycin* or Tunicamycin* or Ureidopenicillin* or Valrubicin* or Vancomycin* or Vaborbactam* or Zorubicin*).mp. [mp=title, abstract, heading word, drug trade name, original title, device manufacturer, drug manufacturer, device trade name, keyword heading word, floating subheading word, candidate term word]	
6	4 or 5	2596281
7	3 and 6	188412
8	Insulin Dependent Diabetes Mellitus/ or Autoimmune Diabetes/ or Insulitis/	152905
9	("type I diabet*" or "type 1 diabet*" or "type one diabet*" or T1D or "juvenile adj2 diabet*" or "p?ediatric diabet*" or "child* adj2 diabet*" or "insulin dependent diabet*" or IDDM or insulitis or "autoimmune diabet*" or "beta cell adj3 auto*" or "beta cell adj3 destruct*" or "beta cell adj3 deplet*" or "beta cell adj3 loss" or "islet adj3 destruct*" or "islet adj3 auto*" or "insulin adj3 auto*" or "insulin adj3 antibod*" or "insulin adj2 deplet*" or "insulin adj2 deficien*" or "insulin adj2 lack*" or "glutamic acid decarboxylase*" or "antiglutamic acid decarboxylase*" or "Glutamate Decarboxylase*" or "antiglutamate Decarboxylase*" or GAD or antiGAD* or GADA or GAD65* or antiGAD65* or "islet antigen 2*" or "IA-2*" or IA2* or IAA or ICA512* or "ICA-512*" or "Tyrosine Phosphatase-like Protein adj3 Auto*" or "Insulinoma Associated 2 adj3 Auto*" or "Zinc Transporter 8 adj2 Auto*" or "Zinc Transporter 8 adj2 antibod*" or ZnT8* or "pancre* adj2 auto*" or "pancre* adj2 antibod*").mp. [mp=title, abstract, heading word, drug trade name, original title, device manufacturer, drug manufacturer, device trade name, keyword heading word, floating subheading word, candidate term word]	549914
10	8 or 9	549914
11	Risk/ or Attributable Risk/ or exp Environmental Risk/ or Risk Assessment/ or Health Risk Assessment/ or Risk Factor/ or Vulnerability/ or Disease Risk Assessment/ or Odds Ratio/ or Incidence/ or Standardized Incidence Ratio/ or prevalence/ or prevalence ratio/	3687132
12	(risk* or odds or ratio or likelihood* or probabil* or chance* or incidence* or prevalen* or rate* or suscept* or vulnerab* or propensit* or associat*).mp. [mp=title, abstract, heading word, drug trade name, original title, device manufacturer, drug manufacturer, device trade name, keyword heading word, floating subheading word, candidate term word]	17943445
13	11 or 12	17944169
14	Juvenile/ or Adolescent/ or Child/ or Infant/ or Baby/ or Preschool Child/ or School Child/ or Toddler/ or Child Development/ or Childhood Disease/ or Child Health/ or Adolescent Development/ or Adolescent Disease/ or Adolescent Health/ or National Longitudinal Study of Adolescent Health/ or Pediatrics/	4260704
15	(child* or baby* or babies* or infan* or toddler* or p?ediatric or preteen* or teen* or adolescen* or "school-age*").mp. [mp=title, abstract, heading word, drug trade name, original title, device manufacturer, drug manufacturer, device trade name, keyword heading word, floating subheading word, candidate term word]	5292727

16	14 or 15	5332136
17	10 and 13 and 16	51160
18	7 and 17	494
**Scopus <Inception to to 2024 August 26>**		
1	TITLE-ABS-KEY((pregnan*) or (gesta*) or (prenatal*) or (“pre-natal*”) or (antenatal*) or (“ante-natal*”) or (perinatal*) or (“peri-natal*”) or (postnatal*) or (“post-natal*”) or (trimester*) or (fetus*) or (foetus*) or (fetal) or (foetal*) or (neonat*) or (newborn*) or (infan*) or (baby*) or (babies*) or (“early life”))	3474796
2	TITLE-ABS-KEY((Antibiotic*) or (“Anti-biotic*”) or (Antibacteria*) or (“Anti-bacteria*”) or (Acetylspiramycin*) or (Aclacinomycin*) or (Aclarubicin*) or (Aculeacin*) or (Afabicin*) or (Alafosfalin*) or (Alalevonadifloxacin*) or (Alamethicin*) or (Allicin*) or (Amfomycin*) or (Amikacin*) or (Aminoglycoside*) or (“Aminopenicillanic acid*”) or (Aminopenicillin*) or (Aminosalicylic Acid*) or (Amoxicillin*) or (Amrubicin*) or (Anisomycin*) or (Annamycin*) or (Ansamycin*) or (Antofloxacin*) or (Aplasmomycin*) or (Aplysianin*) or (Asukamycin*) or (Avibactam*) or (Avilamycin*) or (Azithromycin*) or (Azlocillin*) or (Aztreonam*) or (Azurocidin*) or (Bacampicillin*) or (Bacitracin*) or (Bafilomycin*) or (Balhimycin*) or (Balofloxacin*) or (Beauvericin*) or (Bedaquiline*) or (Bersiporocin*) or (“Beta-lactam*”) or (Betamipron*) or (Bialaphos*) or (Biapenem*) or (Bicozamycin*) or (Bifonazole*) or (Bleomycin*) or (Boromycin*) or (Borrelidin*) or (Brefeldin*) or (Brilacidin*) or (Calcimycin*) or (Carbacephem*) or (Carbapenem*) or (Carbenicillin*) or (Carindacillin*) or (Carumonam*) or (Cefacetrile*) or (Cefaclor*) or (Cefadroxil*) or (Cefalexin*) or (Cefaloridine*) or (Cefalotin*) or (Cefamandole*) or (Cefapirin*) or (Cefatrizine*) or (Cefazedone*) or (Cefazolin*) or (Cefcapene*) or (Cefcanel*) or (Cefditoren*) or (Cefepime*) or (Cefetamet*) or (Cefiderocol*) or (Cefixime*) or (Cefmenoxime*) or (Cefmetazole*) or (Cefminox*) or (Cefodizime*) or (Cefonicid*) or (Cefoperazone*) or (Ceforanide*) or (Cefoselis*) or (Cefotaxime*) or (Cefotetan*) or (Cefotiam*) or (Cefoxitin*) or (Cefpirome*) or (Cefpiramide*) or (Cefpodoxime*) or (Cefprozil*) or (Cefquinome*) or (Cefradine*) or (Cefroxadine*) or (Cefsulodin*) or (Ceftaroline*) or (Ceftazidime*) or (Cefteram*) or (Ceftibuten*) or (Ceftiofur*) or (Ceftizoxime*) or (Ceftobiprole*) or (Ceftolozane*) or (Ceftriaxone*) or (Cefuroxime*) or (Cefuzonam*) or (Cefozopran*) or (Cephacetrile*) or (Cephalosporin*) or (Cephalothin*) or (Cephamycin*) or (Cephradine*) or (Chloramphenicol*) or (Chlortetracycline*) or (Cilastatin*) or (Ciprofloxacin*) or (“Clavulanic Acid*”) or (Clindamycin*) or (Clofazimine*) or (Clarithromycin*) or (Cloxacillin*) or (Concanamycin*) or (Contezolid*) or (Cyclacillin*) or (Cycloheximide*) or (Dactinomycin*) or (Dalbavancin*) or (Dalfopristin*) or (Danofloxacin*) or (Daunomycin*) or (Daunorubicin*) or (Demeclocycline*) or (Delafloxacin*) or (Delamanid*) or (Deoxydoxorubicin*) or (Desmycosin*) or (Dibekacin*) or (Dichloramine*) or (Dicloxacillin*) or (Difloxacin*) or (Dihydrostreptomycin*) or (Dirithromycin*) or (Dioxidine*) or (Doripenem*) or (Doxycycline*) or (Durlobactam*) or (Echinomycin*) or (Edeine*) or (Enmetazobactam*) or (Enniatin*) or (Enoxacin*) or (Eperezolid*) or (Epiroprim*) or (Eravacycline*) or (Erythromycin*) or (Erythronolide*) or (Ethambutol*) or (Ethionamide*) or (Everninomicin*) or (Exebacase*) or (Fidaxomicin*) or (Filipin*) or (Finafloxacin*) or (Fleroxacin*) or (Floxacillin*) or (Fosfomycin*) or (Framycetin*) or (Fusafungine*) or (Fusidate sodium*) or (Fusidic Acid*) or (Funobactam*) or (Garenoxacin*) or (Gatifloxacin*) or (Gemifloxacin*) or (Gepotidacin*) or (Gramicidin*) or (Habekacin*) or (Heliomycin*) or (Hygromycin*) or (Ibezapolstat*) or (Iclaprim*) or (Idarubicin*) or (Ikarugamycin*) or (Imipenem*) or (Isopenicillin*) or (Josamycin*) or (Kanamycin*) or (Ketolide*) or (Lactivicin*) or (Lasalocid*) or	2192133

	(Lascufloxacin*) or (Lefamulin*) or (Leucomycin*) or (Levonadifloxacin*) or (Levofloxacin*) or (Lincosamide*) or (Linezolid*) or (Lincomycin*) or (Lomefloxacin*) or (Lysobactin*) or (Lysostaphin*) or (Lymecycline*) or (Mafenide*) or (Malyngolide*) or (Manumycin*) or (Mecillinam*) or (Meropenem*) or (Mersacidin*) or (Metampicillin*) or (Methacycline*) or (Methicillin*) or (Mepartricin*) or (Mertansine*) or (Metronidazole*) or (Milbemycin*) or (Minocycline*) or (Miraxid*) or (Modithromycin*) or (Moxalactam*) or (Moxifloxacin*) or (Mupirocin*) or (Mureidomycin*) or (Murepavadin*) or (Myxothiazol*) or (Nafcillin*) or (Nafithromycin*) or ("Nalidixic Acid*") or (Nebramycin*) or (Nemonoxacin*) or (Nemorubicin*) or (Neomycin*) or (Neosporin*) or (Netilmicin*) or (Nisin*) or (Nitrofurantoin*) or (Novobiocin*) or (Ofloxacin*) or (Oleandomycin*) or (Oligomycin*) or (Omadacycline*) or (Oritavancin*) or (Oxacillin*) or ("Oxolinic Acid*") or (Oxytetracycline*) or (Pardaxin*) or (Paromomycin*) or (Patulin*) or (Pazufloxacin*) or (Peceleganan*) or (Pefloxacin*) or (Penicillin*) or (Pheneticillin*) or (Pirarubicin*) or (Piperacillin*) or (Pivampicillin*) or (Pivmecillinam*) or (Platensimycin*) or (Plazomicin*) or (Pleuromutilin*) or (Pradimicin*) or (Pretomanid*) or (Pristinamycin*) or (Prulifloxacin*) or (Pyrazinamide*) or (Pyrrolinodoxorubicin*) or (Quinupristin*) or (Quinacillin*) or (Ramoplanin*) or (Ranalexin*) or (Ranbezolid*) or (Ravtansine*) or (Relebactam*) or (Retapamulin*) or (Rifabutin*) or (Rifampin*) or (Rifampicin*) or (Rifamycin*) or (Rifaximin*) or (Roxithromycin*) or (Ruboxyl*) or (Sabarubicin*) or (Saframycin*) or (Salinomycin*) or (Sarecycline*) or (Selamectin*) or (Simocyclinone*) or (Sitafloxacin hydrate*) or (Soravtansine*) or (Squalamine*) or (Streptogramin*) or (Streptomycin*) or (Streptothricin*) or (Sulbactam*) or (Sulbenicillin*) or (Sulfacetamide*) or (Sulfadiazine*) or (Sulfamerazine*) or (Sulfamethoxazole*) or (Sulfamethoxypyridazine*) or (Sulfanilamide*) or (Sulfathiazole*) or (Sultamicillin*) or (Surfactin*) or (Tazobactam*) or (Tebipenem*) or (Telithromycin*) or (Temocillin*) or (Tetracycline*) or (Ticarcillin*) or (Tigecycline*) or (Tinidazole*) or (Tomopenem*) or (Trichostatin*) or (Trimethoprim*) or (Troleandomycin*) or (Tunicamycin*) or (Ureidopenicillin*) or (Valrubicin*) or (Vancomycin*) or (Vaborbactam*) or (Zorubicin*))	
3	1 and 2	179217
4	TITLE-ABS-KEY(("type I diabet*") or ("type 1 diabet*") or ("type one diabet*") or (T1D) or ("juvenile W/2 diabet*") or ("pediatric diabet*") or ("paediatric diabet*") or ("child* W/2 diabet*") or ("insulin dependent diabet*") or (IDDM) or (insulitis) or ("autoimmune diabet*") or ("beta cell W/3 auto*") or ("beta cell W/3 destruct*") or ("beta cell W/3 deplet*") or ("beta cell W/3 loss") or ("islet W/3 destruct*") or ("islet W/3 auto*") or ("insulin W/3 auto*") or ("insulin W/3 antibod*") or ("insulin W/2 deplet*") or ("insulin W/2 deficien*") or ("insulin W/2 lack*") or ("glutamic acid decarboxylase*") or ("antiglutamic acid decarboxylase*") or ("Glutamate Decarboxylase*") or ("antiglutamate Decarboxylase*") or (GAD) or (antiGAD*) or (GADA) or (GAD65*) or (antiGAD65*) or ("islet antigen 2*") or ("IA-2*") or (IA2*) or (IAA) or (ICA512*) or ("ICA-512*") or ("Tyrosine Phosphatase-like Protein W/3 Auto*") or ("Insulinoma Associated 2 W/3 Auto*") or ("Zinc Transporter 8 W/2 Auto*") or ("Zinc Transporter 8 W/2 antibod*") or (ZnT8*) or ("pancre* W/2 auto*") or ("pancre* W/2 antibod*"))	460336
5	TITLE-ABS-KEY((risk*) or (odds) or (ratio) or (likelihood*) or (probabil*) or (chance*) or (incidence*) or (prevalen*) or (rate*) or (suscept*) or (vulnerab*) or (propensit*) or (associat*))	27083608
6	TITLE-ABS-KEY((child*) or (baby*) or (babies*) or (infan*) or (toddler*) or (pediatric) or (paediatric) or (preteen*) or (teen*) or (adolescen*) or ("school-age*"))	6126947

7	4 and 5 and 6	45879
8	3 and 7	402
**Web of Science Core Collection <Inception to to 2024 August 26>**		
1	TS=(pregnan* or gesta* or prenatal* or "pre-natal*" or antenatal* or "ante-natal*" or perinatal* or "peri-natal*" or postnatal* or "post-natal*" or trimester* or f?etus* or f?etal or neonat* or newborn* or infan* or baby* or babies* or "early life")	1936383
2	TS=(Antibiotic* or "Anti-biotic*" or Antibacteria* or "Anti-bacteria*" or Acetylspiramycin* or Aclacinomycin* or Aclarubicin* or Aculeacin* or Afabicin* or Alafosfalin* or Alalevonadifloxacin* or Alamethicin* or Allicin* or Amfomycin* or Amikacin* or Aminoglycoside* or "Aminopenicillanic acid*" or Aminopenicillin* or Aminosalicylic Acid* or Amoxicillin* or Amrubicin* or Anisomycin* or Annamycin* or Ansamycin* or Antofloxacin* or Aplasmomycin* or Aplysianin* or Asukamycin* or Avibactam* or Avilamycin* or Azithromycin* or Azlocillin* or Aztreonam* or Azurocidin* or Bacampicillin* or Bacitracin* or Bafilomycin* or Balhimycin* or Balofloxacin* or Beauvericin* or Bedaquiline* or Bersiporocin* or "Beta-lactam*" or Betamipron* or Bialaphos* or Biapenem* or Bicozamycin* or Bifonazole* or Bleomycin* or Boromycin* or Borrelidin* or Brefeldin* or Brilacidin* or Calcimycin* or Carbacephem* or Carbapenem* or Carbenicillin* or Carindacillin* or Carumonam* or Cefacetrile* or Cefaclor* or Cefadroxil* or Cefalexin* or Cefaloridine* or Cefalotin* or Cefamandole* or Cefapirin* or Cefatrizine* or Cefazedone* or Cefazolin* or Cefcapene* or Cefcanel* or Cefditoren* or Cefepime* or Cefetamet* or Cefiderocol* or Cefixime* or Cefmenoxime* or Cefmetazole* or Cefminox* or Cefodizime* or Cefonicid* or Cefoperazone* or Ceforanide* or Cefoselis* or Cefotaxime* or Cefotetan* or Cefotiam* or Cefoxitin* or Cefpirome* or Cefpiramide* or Cefpodoxime* or Cefprozil* or Cefquinome* or Cefradine* or Cefroxadine* or Cefsulodin* or Ceftaroline* or Ceftazidime* or Cefteram* or Ceftibuten* or Ceftiofur* or Ceftizoxime* or Ceftobiprole* or Ceftolozane* or Ceftriaxone* or Cefuroxime* or Cefuzonam* or Cefozopran* or Cephacetrile* or Cephalosporin* or Cephalothin* or Cephamycin* or Cephradine* or Chloramphenicol* or Chlortetracycline* or Cilastatin* or Ciprofloxacin* or "Clavulanic Acid*" or Clindamycin* or Clofazimine* or Clarithromycin* or Cloxacillin* or Concanamycin* or Contezolid* or Cyclacillin* or Cycloheximide* or Dactinomycin* or Dalbavancin* or Dalfopristin* or Danofloxacin* or Daunomycin* or Daunorubicin* or Demeclocycline* or Delafloxacin* or Delamanid* or Deoxydoxorubicin* or Desmycosin* or Dibekacin* or Dichloramine* or Dicloxacillin* or Difloxacin* or Dihydrostreptomycin* or Dirithromycin* or Dioxidine* or Doripenem* or Doxycycline* or Durlobactam* or Echinomycin* or Edeine* or Enmetazobactam* or Enniatin* or Enoxacin* or Eperezolid* or Epiroprim* or Eravacycline* or Erythromycin* or Erythronolide* or Ethambutol* or Ethionamide* or Everninomicin* or Exebacase* or Fidaxomicin* or Filipin* or Finafloxacin* or Fleroxacin* or Floxacillin* or Fosfomycin* or Framycetin* or Fusafungine* or Fusidate sodium* or Fusidic Acid* or Funobactam* or Garenoxacin* or Gatifloxacin* or Gemifloxacin* or Gepotidacin* or Gramicidin* or Habekacin* or Heliomycin* or Hygromycin* or Ibezapolstat* or Iclaprim* or Idarubicin* or Ikarugamycin* or Imipenem* or Isopenicillin* or Josamycin* or Kanamycin* or Ketolide* or Lactivicin* or Lasalocid* or Lascufloxacin* or Lefamulin* or Leucomycin* or Levonadifloxacin* or Levofloxacin* or Lincosamide* or Linezolid* or Lincomycin* or Lomefloxacin* or Lysobactin* or Lysostaphin* or Lymecycline* or Mafenide* or Malyngolide* or Manumycin* or Mecillinam* or Meropenem* or Mersacidin* or Metampicillin* or Methacycline* or Methicillin* or Mepartricin* or Mertansine* or Metronidazole* or Milbemycin* or Minocycline* or Miraxid* or Modithromycin* or Moxalactam* or Moxifloxacin* or Mupirocin* or Mureidomycin* or Murepavadin* or Myxothiazol* or Nafcillin* or Nafithromycin* or "Nalidixic Acid*" or	1226315

	Nebramycin* or Nemonoxacin* or Nemorubicin* or Neomycin* or Neosporin* or Netilmicin* or Nisin* or Nitrofurantoin* or Novobiocin* or Ofloxacin* or Oleandomycin* or Oligomycin* or Omadacycline* or Oritavancin* or Oxacillin* or “Oxolinic Acid*” or Oxytetracycline* or Pardaxin* or Paromomycin* or Patulin* or Pazufloxacin* or Peceleganan* or Pefloxacin* or Penicillin* or Pheneticillin* or Pirarubicin* or Piperacillin* or Pivampicillin* or Pivmecillinam* or Platensimycin* or Plazomicin* or Pleuromutilin* or Pradimicin* or Pretomanid* or Pristinamycin* or Prulifloxacin* or Pyrazinamide* or Pyrrolinodoxorubicin* or Quinupristin* or Quinacillin* or Ramoplanin* or Ranalexin* or Ranbezolid* or Ravtansine* or Relebactam* or Retapamulin* or Rifabutin* or Rifampin* or Rifampicin* or Rifamycin* or Rifaximin* or Roxithromycin* or Ruboxyl* or Sabarubicin* or Saframycin* or Salinomycin* or Sarecycline* or Selamectin* or Simocyclinone* or Sitafloxacin hydrate* or Soravtansine* or Squalamine* or Streptogramin* or Streptomycin* or Streptothricin* or Sulbactam* or Sulbenicillin* or Sulfacetamide* or Sulfadiazine* or Sulfamerazine* or Sulfamethoxazole* or Sulfamethoxypyridazine* or Sulfanilamide* or Sulfathiazole* or Sultamicillin* or Surfactin* or Tazobactam* or Tebipenem* or Telithromycin* or Temocillin* or Tetracycline* or Ticarcillin* or Tigecycline* or Tinidazole* or Tomopenem* or Trichostatin* or Trimethoprim* or Troleandomycin* or Tunicamycin* or Ureidopenicillin* or Valrubicin* or Vancomycin* or Vaborbactam* or Zorubicin*)	
3	1 and 2	43943
4	TS=(“type I diabet*” or “type 1 diabet*” or “type one diabet*” or T1D or “juvenile NEAR/2 diabet*” or “p?ediatric diabet*” or “child* NEAR/2 diabet*” or “insulin dependent diabet*” or IDDM or insulitis or “autoimmune diabet*” or “beta cell NEAR/3 auto*” or “beta cell NEAR/3 destruct*” or “beta cell NEAR/3 deplet*” or “beta cell NEAR/3 loss” or “islet NEAR/3 destruct*” or “islet NEAR/3 auto*” or “insulin NEAR/3 auto*” or “insulin NEAR/3 antibod*” or “insulin NEAR/2 deplet*” or “insulin NEAR/2 deficien*” or “insulin NEAR/2 lack*” or “glutamic acid decarboxylase*” or “antiglutamic acid decarboxylase*” or “Glutamate Decarboxylase*” or “antiglutamate Decarboxylase*” or GAD or antiGAD* or GADA or GAD65* or antiGAD65* or “islet antigen 2*” or “IA-2*” or IA2* or IAA or ICA512* or “ICA-512*” or “Tyrosine Phosphatase-like Protein NEAR/3 Auto*” or “Insulinoma Associated 2 NEAR/3 Auto*” or “Zinc Transporter 8 NEAR/2 Auto*” or “Zinc Transporter 8 NEAR/2 antibod*” or ZnT8* or “pancre* NEAR/2 auto*” or “pancre* NEAR/2 antibod*”)	166642
5	TS=(risk* or odds or ratio or likelihood* or probabil* or chance* or incidence* or prevalen* or rate* or suscept* or vulnerab* or propensit* or associat*)	21189802
6	TS=(child* or baby* or babies* or infan* or toddler* or p?ediatric or preteen* or teen* or adolescen* or “school-age*”)	3418248
7	4 and 5 and 6	19625
8	3 and 7	72
